# Clinical outcomes of intermittent panitumumab based-therapy for previously treated older patient with metastatic colorectal cancer: a case report and review of literature

**DOI:** 10.3389/fonc.2024.1369952

**Published:** 2024-04-03

**Authors:** Gerardo Rosati, Luigi Annunziata, Enrico Scarano, Francesca Dapoto, Domenico Bilancia

**Affiliations:** ^1^ Medical Oncology Unit, “S. Carlo” Hospital, Potenza, Italy; ^2^ Department of Radiology, “S. Carlo” Hospital, Potenza, Italy; ^3^ Clinical Trial Center, “S. Carlo” Hospital, Potenza, Italy

**Keywords:** case report, panitumumab, reintroduction, intermittent strategy, colorectal cancer, older patient

## Abstract

**Background:**

Metastatic colorectal cancer is one of the most common causes of cancer death worldwide, and its incidence increases with age. Treating an older RAS and BRAF wild-type patient represents a challenge for the medical oncologist, even more so for those patients defined as “vulnerable” and undergoing at least two lines of therapy. In this context, recent evidence supports the role of retreatment with anti-EGFR inhibitors and the use of liquid biopsy. However, frequent skin toxicity constitutes a limitation of therapy, especially in older people. Since it has been described that continuous administration of these monoclonal antibodies leads to acquired resistance to anti-EGFRs, with consequent therapeutic failure, an intermittent strategy with chemotherapy plus an anti-EGFR could help maintain the efficacy of the treatment over time, delaying the resistance and improving patients’ quality of life.

**Case presentation:**

In this case report, we describe the case of an older RAS and BRAF wild-type patient reporting a clinical response after first-line chemotherapy with FOLFOX + panitumumab, subsequently interrupted in the absence of disease progression. After radiological worsening and two additional lines of therapy, the reintroduction of panitumumab plus 5-fluorouracil, administered with a stop-and-go strategy, allowed the patient to benefit from the same drugs for 2 years from diagnosis, to achieve a clinical response during fourth-line treatment lasting more than 3 years, to delay resistance and to avoid unacceptable anti-EGFR skin toxicity. This patient, who died from a myocardial infarction more than 5 years after diagnosis, represents the case of a good synergy between molecular profile of disease and reintroduction of an anti-EGFR with intermittent strategy.

## Introduction

Colorectal cancer (CRC) is the third most common cancer in the world, especially widespread in western countries and results from a variety of risk factors, such as sedentary lifestyle, alcohol and tobacco abuse, obesity and incorrect eating habits, deriving from the low intake of fruit and vegetables and vice versa the excessive consumption of red meat, fatty and processed foods (1, 2).

As the incidence of CRC increases with age and populations are getting older (3, 4), the interest in knowing how to treat an older metastatic patient is growing. These patients consist of an extremely varied population of subjects, ranging form fit to frail, characterized by various clinical conditions, few or many comorbidities and different ability to tolerate chemotherapy (5). The implementation of a multidimensional geriatric assessment (CGA) and accurate tools such as the Geriatric-8 (G8) and the Vulnerable Elders Survey-13 (VES-13) will be able to guide the clinician on a case-by-case basis in his decision-making process (6).

The complexity and the best treatment approach for each patient suffering from metastatic CRC (mCRC) are not established only by considerations of his age, but by several prognostic and predictive factors such as performance status (PS), comorbidities, degree of diffusion and locations of disease, its biomolecular profile and analysis of any eventual previous line of therapy (7).

Limited to pre-treated patients, two historic phase III studies (CORRECT and RECOURSE) demonstrated that two oral agents, regorafenib and trifluridine-tipiracil, improve progression-free survival (PFS) and overall survival (OS) in those who have already received fluoropyrimidines, oxaliplatin, irinotecan and biological drugs (8, 9). Moreover, a quite recent phase III study, which randomized nearly 500 subjects with the same characteristics, demonstrated that adding bevacizumab to trifluridine-tipiracil resulted in longer OS than trifluridine-tipiracil alone [hazard ratio (HR) for death, 0.61] (10). Finally, a few months ago in another international, randomized, double-blind, placebo-controlled, phase III study (FRESCO-2), fruquintinib, a highly selective and potent oral inhibitor of vascular endothelial growth factor receptors 1-3, obtained a significant and clinically meaningful benefit in OS compared with placebo in patients with refractory mCRC (HR 0.66) (11).

Nevertheless, reintroduction or rechallenge of the effective first-line therapy is at present a strategy with great clinical interest in third or fourth-line, particularly when it includes anti-epidermal growth factor receptor (EGFR) monoclonal antibodies (cetuximab and panitumumab) (12). In this sense, various strategies and combinations of chemotherapeutic agents have been tested (13–15). A pooled analysis from four phase II studies have underlined how helpful the use of liquid biopsy can be in the selection of patients for rechallenge with EGFR inhibitors (16). However, rechallenge must be differentiated from reintroduction, defined as the administration of a therapy with which the patient has experienced previous clinical benefit and that had been discontinued without disease progression (PD).

Recently, a phase II randomized trial has demonstrated that the an intermittent therapy based on anti-EGFRs limits toxicity and produces a long PFS without any detrimental effect on OS (17). This approach could be particularly attractive in pre-treated older patients, especially among those who require adaptive treatment, as in vulnerable subjects to whom many of them belong anyway (6).

In this context, by presenting a clinical case of a vulnerable older mCRC patient, wild-type (wt) for rat sarcoma virus (RAS) and B-rapidly accelerated fibrosarcoma (BRAF) oncogenes, and re-treated in the fourth-line with panitumumab plus 5-fluorouracil (5-FU), we review the variables as well as the potential of a therapy administered according to a stop and go strategy, three months on and three months off.

## Case presentation

Our patient, R.M.S., born in 1935 and at the age of 81, following repeated episodes of rectal bleeding, went to the emergency room in September 2016. Having collected the anamnesis, he was admitted to surgery for further tests. After verifying its comorbidities (ischemic heart disease, benign prostatic hypertrophy and osteoarthritis) with a moderate risk attribution of 4 according to the Charlson comorbidity index (CCI) (18), a colonoscopy was performed with diagnosis of an ulcerative-vegetating lesion affecting the sigmoid. A subsequent biopsy confirmed an adenocarcinoma. The staging of the disease with computed-tomography (CT) showed no secondary lesions and therefore surgeons proceeded to segmental resection of the involved colonic tract with histological diagnosis of moderately/poorly differentiated infiltrating adenocarcinoma and widespread involvement in 4 of the 18 lymph nodes removed. In view of age and absence of distant metastases, the patient underwent adjuvant chemotherapy with capecitabine for 6 months, without significant toxicities.

**Figure 1:**
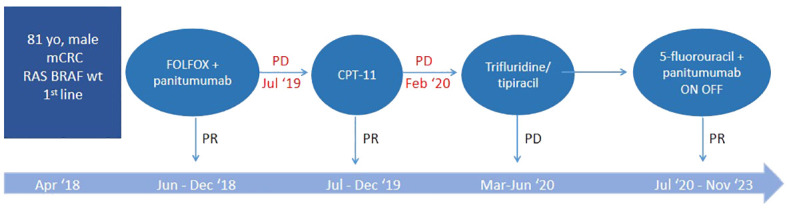
Specific time points corresponding to the therapeutic process. PR, partial response; PD, progressive disease.

In April 2018, at the age of 83, during follow-up, our subject underwent a CT scan which highlighted multiple liver metastases, located in the VI, VII and VIII segments, with the largest having a diameter of 35 mm. The patient, mainly due to his comorbidities, was not candidate for surgery and the molecular determination of RAS and BRAF did not highlight any mutations. After evaluation with the G8 and performing the CGA, the patient was classified as vulnerable. Therefore (specific time points in **Figure 1**), a first-line treatment was undertaken with chemotherapy according to the FOLFOX6 [oxaliplatin, leucovorin (LV) and 5-fluorouracil (5-FU)] regimen at doses reduced by 20% plus panitumumab starting from June up to December for a total of 12 cycles (of which the last three with the anti-EGFR antibody alone), obtaining a partial response (PR) documented by CT. Therapy was discontinued in the absence of PD following the patient’s will as well as grade 2 conjunctivitis, grade 3 maculopapular rash, and grade 2 paronychia. Conversely, hematological toxicity was moderate and did not complicate regular administration of cycles of therapy.

In July 2019 a CT highlighted liver PD. Second-line chemotherapy with irinotecan was then undertaken, without 5-FU to avoid excessive hematological toxicity and without bevacizumab, taking into account the patient’s cardiac comorbidities. The therapy was administered for 12 cycles with modest toxicities, obtaining a PR.

**Figure 2:**
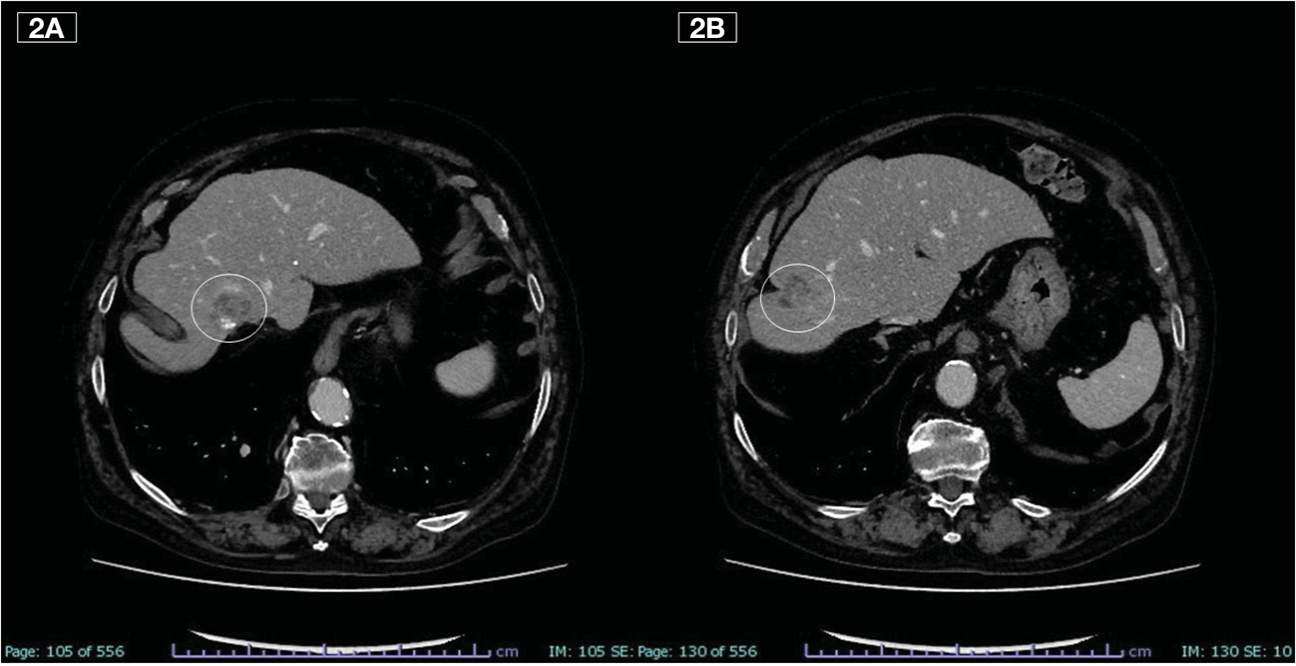
Liver metastases **(A, B)** at the beginning of fourth-line therapy (July 2020).

In February 2020, the CT scan showed a new liver PD. A third-line treatment was proposed with an oral drug such as trifluridine/tipiracil which the patient received from March to June for a total of 3 cycles. The subsequent CT scan, however, showed rapid PD (**Figures 2A, B**) and treatment was therefore definitively discontinued.

**Figure 3:**
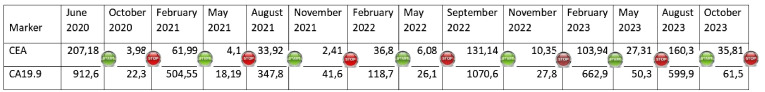
Tumor marker levels and therapy decision making.

At this point, the patient was candidate for a fourth-line therapy with reintroduction of panitumumab plus 5-FU. The treatment began in July and, after the first 3 months, a total normalization of tumor marker levels was observed. Furthermore, taking into account that our subject was now 85 years old, having assessed his comorbidities, it was deemed appropriate to adopt an intermittent therapeutic strategy, characterized by treatment intervals of 3 months (6 cycles) followed by a 3-month stop in order to limit potential toxicities. This would also have resulted in fewer hospital visits for both the patient and the caregiver, helping to improve their quality of life (QoL). Therapy and its suspension were simply guided by the trend of tumor markers, CEA and Ca 19.9 (**Figure 3**).

**Figure 4:**
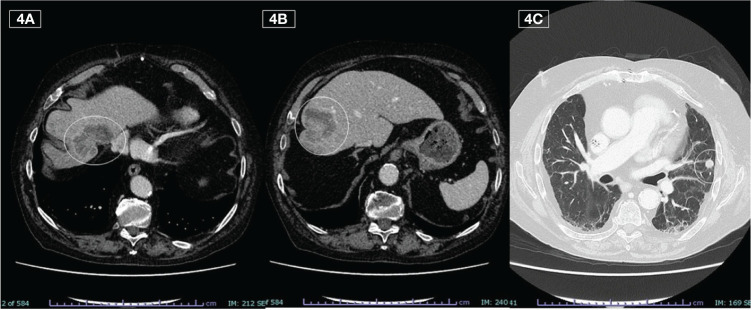
Liver **(A, B)** and lung **(C)** metastases detected on last CT during the off phase (September 2023)

A CT scan, performed in September 2023, highlighted hepatic and pulmonary PD, but it was only apparent because found during the planned suspension of therapy (**Figures 4A-C**). The toxicities reported were modest, characterized by conjunctivitis and grade 2 maculopapular skin rash, easily controlled with topical products and minocycline, if necessary.

After resuming therapy with further 6 cycles, the tumor markers once again decreased and treatment was suspended. During this phase, 42 months after the start of the fourth line, the patient, at the age of 88, died on December 14th following myocardial infarction.

## Discussion

The survival of patients with mCRC, mainly if RAS and BRAF wt, has increased significantly in recent years, exceeding 30 months. This has been achieved by adopting various strategies such as continuum of care, resection of metastatic disease, local ablative treatments, the contribution of molecular biology and target therapies, and rechallenge with drugs previously used. This could also be the case for many older patients when treated with previous lines of regimens containing oxaliplatin, irinotecan, fluoropyrimidines, antiangiogenic and anti-EGFRs inhibitors, especially if they maintain a good PS and are candidates for therapies beyond the second-line.

In this situation, rechallenge with anti-EGFRs could represent a valid clinical option. It consists in the re-administration of a cetuximab or panitumumab based-treatment to which the tumor has developed resistance. The application of this strategy presupposes that patients should have obtained a major response during first-line chemotherapy combined with an anti-EGFR drug and should have received a second-line therapy after PD. The time between the end of first-line therapy and the start of third-line therapy should have been at least 4 months. But most of all, an analysis of RAS/BRAF wt circulating tumor DNA (ctDNA) by liquid biopsy is necessary because it represents the only biomarker that could identify patients that are potentially benefiting from anti-EGFR rechallenge. Thus, an individualized patients’ data pooled analysis (16) responding to these characteristics calculated OS, PFS, disease control rate (DCR) and overall response rate (ORR) for 114 cases enrolled in the CAVE, VELO, CRICKET and CHRONOS trials (19–22). Median PFS and median OS were 4.0 months and 13.1 months, respectively, while the ORR was 17.5% (20/114) and the DCR was 74.6% (85/114). Almost one out of three patients significantly benefited from anti-EGFR rechallenge therapy (6-months PFS rate, 32.5%; 18-months OS rate, 31.6%).

However, as mentioned above, the reintroduction of a therapy responds to different criteria. In our case, the first-line treatment was discontinued after obtaining a PR, in absence of PD. Typically, this happens in clinical practice either due to toxicity or according to the patient’s will or when the physician believes that indefinite administration of the therapy will not bring about any further benefit (23, 24). These three conditions were all found in our patient. Although the treatment was well tolerated from a hematological point of view, the skin toxicity and photophobia resulting from grade 2 conjunctivitis determined the patient’s will to ask for its suspension. On the other hand, older people are more focused on their QoL and on therapies that can lead to tangible symptomatic improvement rather than cyclically undergoing prolonged treatments which can be toxic, and cause health issues both to the patient and to caregivers (25).

Note that the re-proposal of panitumumab and 5-FU in the fourth- rather than third-line of therapy was conceived by the need to reduce hospital admissions to our patient at the beginning of the Sars-Cov-2 viral pandemic by resorting to an oral drug such as trifluridine/tipiracil. Indeed, a meta-analysis with over 611,000 subjects from China, Italy, Spain, United Kingdom, and New York State has reported the effect of age on mortality in patients with COVID-19 and the highest rate was observed in octogenarians (26).

It has been demonstrated that RAS mutantions emerge dynamically in ctDNA during anti-EGFR therapy and decline when treatment is suspended (27). In particular, relative mutant allele frequency decays exponentially with a cumulative half-life of 4.4 months (28). This could lead to better identification of those cases susceptible to maintaining a blockade with anti-EGFR or reintroducing it after a pressure-free pause period ensuring treatment-free windows with undoubted benefit on patients’ QoL. To reinforce these theories, a *post hoc* analysis of the VALENTINO trial demonstrated that when patients in PD and not in treatment, underwent conventional second-line chemotherapy or reinduction with anti-EGFR (all patients had an anti-EGFR-free interval of at least 3 months), a similar result in terms of PFS was achieved in the latter, but with a significantly longer OS and a higher ORR than in the former (29). In this scenario, we recall the positive results of the aforementioned randomized IMPROVE study which has PFS in treatment (PFS_OT_) as primary endpoint (16). The intermittent administration of FOLFIRI (irinotecan, LV and 5-FU) plus panitumumab until progression rather than continuous showed that the median PFS_OT_ was 12.6 months in the continuous arm and 17.6 months in the intermittent arm, with 1-year PFS_OT_ rates of 51.7% and 61.3%, respectively.

In conclusion, underlining that to the best of our knowledge there are no similar cases reported in the literature, an effective intermittent reintroduction of an anti-EGFR based-therapy in the fourth-line, without resorting to liquid biopsy in consideration of the wide interval from the end of the first-line interrupted in the absence of PD, allowed us to continue the same treatment for a unexpected time of over 3 years with great advantage for the patient both in terms of OS and QoL.

## Data availability statement

The raw data supporting the conclusions of this article will be made available by the authors, without undue reservation.

## Ethics statement

Ethical approval was not required for the studies involving humans because the manuscript reports a rare case and not data from a clinical study. The study was conducted in accordance with the local legislation and institutional requirements. The participants provided their written informed consent to participate in this study. Written informed consent was obtained from the individual(s) for the publication of any potentially identifiable images or data included in this article.

## Author contributions

GR: Writing – review & editing, Writing – original draft, Visualization, Validation, Supervision, Resources, Investigation, Funding acquisition, Formal analysis, Data curation, Conceptualization. LA: Writing – review & editing, Visualization, Validation, Supervision, Formal analysis, Data curation, Conceptualization. ES: Writing – review & editing, Visualization, Validation, Supervision, Software, Data curation. FD: Writing – review & editing, Validation, Supervision, Software, Data curation. DB: Writing – review & editing, Validation, Supervision, Resources, Methodology, Funding acquisition, Formal analysis.
